# Microaggressions and Coping with Linkages for Mentoring

**DOI:** 10.3390/ijerph18115676

**Published:** 2021-05-26

**Authors:** Nisha Nair, Deborah Cain Good

**Affiliations:** Katz Graduate School of Business, University of Pittsburgh, Pittsburgh, PA 15260, USA

**Keywords:** microaggressions, mentoring, minority identity, coping mechanisms

## Abstract

Microaggressions can have damaging health impacts on minority groups experiencing exclusion through such forms of discrimination and bias. Using focus groups of different marginalized groups and through in-depth interviewing, we analyze the ways in which marginalized identities respond to and deal with microaggressions and highlight some relevant linkages to mentoring. Through a qualitative analysis of microaggression experiences, along the lines of race, gender, sexual orientation, and religion, we explore different coping mechanisms and potential linkages to mentoring. Our results indicate some underlying patterns of sense-making, categorized as coping by (a) resisting or reclaiming their voice, (b) retreating, reframing, or withdrawing, (c) rejecting or stonewalling, (d) restraining and internalizing, (e) seeking support and reconnecting (with safe spaces), and (f) redoubling (effort). For each of the coping strategies discussed, we also identify and advance mentoring linkages in the context of coping with microaggressions.

## 1. Introduction

There is an increasing understanding today that discrimination and exclusion can play out in alternate and subtle forms, such as that of microaggressions [1,2,3,4,5,6,7]. Some have even characterized the microaggression experience as worse than blatant racism [8], where the expression of exclusion and denigration, albeit subtle, can have damaging impacts on the mental and physical health of the recipient of the aggression [1].

Early research on microaggressions, driven largely through the pioneering work of Sue and colleagues [2,9], conceptualized microaggressions as “brief and commonplace daily verbal, behavioral, and environmental indignities, whether intentional or unintentional, that communicate hostile, derogatory, or negative racial slights and insults to the target person or group” ([2], p. 273). Such forms of exclusion and slights, which began with work on racial microaggressions, have since been extended to other kinds of marginalization, operating at the level of gender [10], sexual identity [11], ethnic and other minority identities [7,12], and even religion [13]. Increasingly, microaggressions are being studied using an intersectional framework as well, such as that of gendered racial microaggressions [14].

A number of studies have reported the harmful psychological, emotional, and physical health impacts of microaggressions [2]. Effects of microaggressions have been linked to traumatic stress [5], sleep disorders, experiences of negative effects [15], and even expressions of violence [16]. The influence of racial microaggressions on mental health has received particular attention and has been linked with mental health problems in different studies. In one study by Torres, Driscoll, and Burrow [17], racial microaggressions were found to be associated with greater perceived stress and linked to greater depressive symptoms among highly achieving African Americans. Reporting on the microaggressions experienced by Black males in historically White research institutions, Smith et al. [18] discussed the effects of racial battle fatigue, with accompanying emotions of frustration, anger, disappointment, resentment, anxiety, helplessness, and fear.

### Coping and Mentoring

If microaggressions have the potential to exert harmful effects on the recipients’ mental and physical health, then approaches to coping with microaggressions also become central in managing the effects of microaggressions. The psychological distress experienced by Black Americans in the face of microaggressions has been referred to as the psychological consequences of feeling powerless, invisible, and experiencing pressure to conform and represent one’s group [19]. Looking at the intersection of race and gender, Lewis and colleagues [20] examined some of the strategies that Black women use to cope with microaggressions, which included resistance coping strategies, such as using one’s voice to push back, and collective coping strategies, such as leaning on one’s support network. Active coping was also found to moderate the relationship between racial microaggressions and perceived stress, such that individuals who engaged in active coping were found to report lower stress levels [17]. Elsewhere, coping with microaggression was hypothesized as a mediator of the relationship between racial microaggressions and depressive symptoms [21]. The authors also tested a moderator relationship, with coping moderating the relationship between gendered racial microaggressions and the experienced depression. Their results indicated that coping in the form of disengagement was a significant mediator of the relationship between microaggressions and depression. Disengagement coping strategies were found to be associated with an increase in gendered racial microaggressions, which, in turn, were associated with increased depressive symptoms. This points to the potential for the use of alternative interventions or strategies such as mentoring as a means to coping in order to ameliorate the effects of the experienced microaggressions.

Mentoring has been known to have positive effects or outcomes, particularly for minority identities. In a study [22] examining the effect of various factors influencing Latino students’ retention in high school, mentoring was identified as one of the factors impacting the successful completion of high school through its effect on remediation and empowerment processes. Castellanos and colleagues have highlighted the important role that mentoring plays regarding the experience of racial and ethnic minority undergraduate students in their study report on the effect of mentoring driving the college satisfaction of minority students [23]. Other studies have reported on the effect of mentoring on the facilitation of women’s careers to leadership positions. In a study on Asian American women’s pathways to school leadership, it was found that gendered and racial expectations were navigated by the women through mentorship support, which was instrumental in their ascension to leadership positions [24]. An alternative viewpoint posits mentoring as facilitating resistance to exclusion. In his phenomenological research on minority faculties in higher education, Casado Pérez [25] discussed the resistance approach to mentorship programs, where mentoring can facilitate resistance and offer aid to confront institutional forms of exclusion and discrimination.

There is evidence that mentoring can facilitate coping in the face of discrimination and exclusion. Prior research has established the efficacy of mentoring in facilitating coping when experiencing racial discrimination with regard to low-income Latina youth. In their quantitative model exploring mentoring as a mediator or moderator of the association between racial discrimination and coping efficacy, Sánchez and colleagues [26] found that higher mentoring quality was significantly associated with higher coping efficacy. In a study exploring coping responses to race-related stressors of Black undergraduate students attending predominantly White institutions, Griffith and colleagues [27] pointed to the role of natural mentors, who are identified as nonparental adults from the students’ preexisting social networks, in mentoring and facilitating the coping process. Elsewhere, natural mentoring relationships [28] were found to aid student adjustment to college life. There is also support for the use of peer mentoring in other domains as a means of coping. Imogen [29] studied a particular intervention using peer mentors to see if it could help with reducing students’ mathematical anxiety. Mentors, in this case, provided encouragement and demonstrated skills to cope with being ‘stuck’ in order to build mathematical resilience and reduce anxiety.

Given the role that mentoring can play in countering discrimination and exclusion, with regard to microaggressions as well, mentoring has the potential to aid in coping with the microaggression experience. One of the themes surfacing in research on racial microaggressions experienced by Black faculty members in counseling programs was the lack of mentoring received by those experiencing microaggressions [30]. Examining the microaggression experience for Black men in predominantly White organizations, Pitcan and colleagues [31] observed that both internal and external mechanisms of coping were employed by the Black men in the aftermath of experienced microaggressions. These included employing social networks as an external mechanism and compartmentalization as an internal mechanism to buffer and counter the experience of microaggressions. Focusing on Black women managers in the corporate world [32], the researchers conducted interviews with women in senior-level corporate positions in America, uncovering racial microaggression themes of stereotyping, invisibility, and exclusion, among others. Employing support networks and seeking sponsorship and mentoring were identified as coping strategies, along with techniques like armoring and turning to religion and spirituality. Some of the other adaptive responses used by people of color to cope with racial microaggressions in their professional lives have been reported to be confrontation as well as seeking out support and mentoring, among other strategies [33]. Both adaptive and maladaptive coping strategies have been reported [34] as ways of dealing with racial microaggressions. One of the ways to deal with the stressors of experiencing racial microaggressions for Blacks in predominantly White institutions has also been posed as turning to the African American student network on college campuses [35]. In separate research [36], it is argued that women of color, being traditionally marginalized in areas of science, technology, engineering, and mathematics (STEM), are more susceptible to the experience of microaggressions. Using interview data from women of color in STEM, the authors alluded to the idea of counterspaces, both physical and conceptual ones, that can operate in a variety of ways, such as the cultivation of mentoring relationships and peer-to-peer mechanisms of support. Such counterspaces are thought to harbor a safe space for marginalized groups, facilitating a mechanism to counter exclusion.

Since microaggressions have been known to have damaging effects on the mental and physical health of minority identities, given that mentoring has been known to aid in coping with the experience of exclusion, in this paper, we draw linkages between microaggressions, coping, and mentoring. We analyze the coping mechanisms employed by different minority identities, along the lines of race, gender, sexual orientation, and religion, in dealing with a microaggression experience and extend the results of our study to forward linkages for mentoring as a means to counter or cope with the microaggression experience.

## 2. Materials and Methods

To understand the mechanisms of coping that recipients of microaggressions utilize, we followed a qualitative phenomenological approach to uncover the lived experiences of microaggressions. We employed a methodology of detailed focus group discussions and interviews with participants of different marginalized identity groups. Since focus groups [37] are particularly useful for obtaining in-depth information on concepts such as microaggression and the responses to it, we employed four different focus groups, each focusing on gender-, race-, religion-, and sexual-orientation-related microaggressions, respectively. Sue [6] affirms that microaggressions are best understood through the lived experience of the participants themselves, and this is in line with recent calls [4] to consider the subjectivities of participants in understanding the microaggression experience. We followed up the focus groups with detailed interviews to fully understand the sense-making process of dealing with microaggressions.

All study participants were students at a large Midwestern university in North America. There was a total of 21 participants in our focus groups and 11 participants in our follow-up interviews. Each focus group focused on the microaggressions experienced by a specific marginalized identity group. Thus, the four focus groups were centered around gender- (n = 7), race- (n = 7), religion- (n = 4) and sexual-orientation-related (n = 3) microaggressions. This was followed by in-depth interviews with 11 participants, with subjects identifying as belonging to more than one minority identity and having experienced microaggressions along any of their marginalized identities. This also allowed us to explore the intersectional [38] microaggression space and examine in further detail the coping mechanism employed. The average age of participants was 28 years; 61% were graduate students, and 39% were undergraduates; the majority of our participants were female (70%), while 30% were male.

We used a semistructured interview protocol, adapting Sue et al.’s [2] interview guide on microaggressions, for both the focus groups and the interviews. Participants in our study were given a research brief beforehand that outlined the purpose of the study (the present study was part of a larger study [39] exploring the nature and forms of microaggressions in addition to the coping and sense-making processes employed). For each focus group and the interviews, we began by describing what a microaggression is and invited participants to share their microaggression experiences along with their coping approaches. We followed up with questions probing how participants processed the microaggression experience in terms of their cognitive, emotional, and behavioral responses to the microaggressions. All questions were kept open-ended to facilitate further dialogue. Sample questions included: “Have you ever been in a situation where you may have felt invalidated, snubbed, denigrated, or discriminated against in some form, owing to your minority identity?”, “How did you feel or react to the microaggression?”, and “What helped you deal with the microaggression, or how did you cope with the experience?”.

The sessions for both the focus groups and interviews varied in duration from 60 min to about 120 min. All sessions were facilitated by one of the authors, and the sessions were audio-taped with the subjects’ permission to be later transcribed, removing any identifier information before the data analysis. The study was funded by the University Research Council’s funding on diversity, and all subjects were compensated for participation through student WePay cards, receiving USD 40 for the focus groups and USD 50 for interview participation. Students were invited to participate in the study through advertisements in student clubs and mail sent out through the respective program offices to the broader student community. Informational mail about the study was also sent out by the authors to students in their classes to spread the word and solicit participation in the study.

For analyzing the data, we followed a phenomenological approach, according to the guidelines of Moustakas [40] and Creswell [37]. Accordingly, we started by transcribing all the focus group and interview recordings and then engaging in a detailed reading and rereading of the transcripts in order to get a sense of the whole. The next step was extracting relevant statements to develop central themes and subthemes to integrate the themes into the larger description of the phenomenon under exploration. All transcripts were read independently by the researchers, and the themes were then identified collaboratively.

## 3. Results

Analysis of our focus group and interview transcripts revealed some underlying thematic areas of coping with microaggressions. In the following sections, we discuss each of these emergent approaches to coping, along with relevant excerpts from varied microaggression foci or marginalized identity categorizations. We also put forward linkages for mentoring where relevant.

### 3.1. Coping Strategies

Our analysis uncovered six different coping strategies employed by the different marginalized groups. Each of the sense-making strategies is discussed below.

#### 3.1.1. Coping by Resisting and Reclaiming One’s Voice

This form of coping refers to the broad set of approaches that include offering up resistance of some sort in the face of a microaggression. The resistance can be in the form of countering or calling out the microaggression, being vocal or verbalizing the perceived slight in relation to the microaggression, reclaiming a voice and expressing solidarity with the marginalized identity targeted in the course of the microaggression, or even educating the perpetrator of the aggression on what is offensive, exclusionary, or discriminatory about the microaggression in the hope of countering such forms of aggression. Such forms of resistance are evidenced in the following excerpts:

*“I think I can remember a situation at my last job where somebody assumed a stereotype, I forgot what the context was, but was assuming a stereotype about Black people and I chose to tell them like, listen, that’s not the case. Like you need to widen your understanding of that.”*

*“If I experience a microaggression, I would be more likely to make sure that person knows that I didn’t appreciate that. So… when somebody would say… “ Well, did you hear the latest hip hop?” I would say, “No, not yet, I’m not really into it!”*

The above quotes refer to microaggressions when stereotypes about Black people are projected in everyday conversations, knowingly or unknowingly, and this is perceived as a slight because it invalidates the whole self and renders the individual that the microaggression is directed at almost invisible when viewed through the singular prism of race.

*“If a person doesn’t have any verbal restraint when they were talking to me, then I don’t have to hold my punch. So somebody says, oh, you like watermelon? I could say no I don’t! I really don’t, but it’s just like I’m direct with it. Using a very quick, succinct, NO, I don’t like that or, no, I don’t really subscribe to that.”*

Similar forms of rebuttal were observed in relation to gender and sexual orientation as well. For individuals subscribing to a nonheteronormative orientation, assumptions such as being gay is being attracted to every other person of the same gender or that bisexual women are comfortable with threesomes were also met with some form of resistance, as the following excerpt suggests:

*“So I typically just call it out and say, why would you say that? I don’t think that’s an appropriate thing you say. I’m pretty assertive that way. I don’t think most people I know would call out those things, but I’ve kind of made a mission to bring awareness to it because people don’t know.”*

*“There’s two schools of thought. Some people say that I won’t respond to ignorance of people, and other people that say, no, I want to correct. I tend to sway going towards the correction because I want them to have 100% understanding that what you said was wrong!”*

One form of resistance was articulated as reaffirming the particular identity that is being targeted by a microaggression. This was most observed in the context of race.

*“For me, I’m not giving up, I’m not forfeiting my Blackness. Anybody comes and they say something wrong with being Black or they say something discriminatory or derogatory, that moment I’ll lose myself and lose who I am as a person. I think some people that are Black and Brown tend to forfeit that; I’m just going to simply be a part of that culture to be accepted, or the fact of the matter is, to me, some people would never, ever accept you. If I assimilate, they are still not going to accept me, so I’m better off being myself to the fullest degree because that’s what’s right. And if they can’t deal with it, then that’s their problem.”*

Microaggressions directed at religion evoke a similar response where the affronted party resists the marginalization by attempting to educate the offender through an explanation of the basic tenets of the religion.

*“Like about the whole Catholic church, the scandal I’ll say, this totally is not what we’re teaching as a church. We say it in mass, every single week during the homily, it’s like this whole thing that’s happened is absolutely terrible. It’s the act of the devil, you know, this is not what it’s like to be a Catholic. It’s mostly developing ways to talk and communicate to other people that they might understand or be more accepting of it.”*

As stated in the above quotes, a microaggression that denigrates a marginalized identity can lead the recipient to question that particular identity or “lose themselves” in the process. It is here where mentoring can play a role in helping individuals reaffirm and strengthen kinship with their marginalized identities when the microaggression experience leaves them feeling vulnerable. Additionally, while some respondents spoke about calling out the microaggressions, not all respondents adopted similar strategies in the face of a microaggression. Even for those who may be predisposed to resisting or countering a microaggression, mentoring can help with evolving ways to call out common forms of microaggressions.

#### 3.1.2. Coping by Retreating, Reframing, or Withdrawing

While calling out a microaggression is one form of coping, withdrawing, ignoring, or disassociating from the context, situation, or individual directing the microaggression is the opposite. It is a mechanism borne out of the need for self-preservation or protecting oneself from further harm or exposure. It emerges from a sense of discomfort engendered in the recipient of the microaggression, where there is some kind of assumption, othering, or invalidation taking place, either intentionally or otherwise.

For someone subscribing to a minority religion or who identifies as an atheist, it can be about conversations about faith, as the following quotes suggest:

*“Like when they’re talking about religion, I never participate in the conversation.”*

*“And I remember this one day I was just on Facebook and my Girl Scouts leader had actually shared this photo and it was towards Muslim people, but it didn’t explicitly state it, and then it made me feel awful. And I actually stopped going to girls scouts after that.”*

Similar mechanisms of coping by separating or disassociating from people who may be perceived as racist, sexist, or homophobic were also observed.

*“And so I wanna dissociate myself from those people. I want to be around good people, doesn’t matter what their gender is. Yes, redirect my energy towards good people.”*

*“I would say that I distance myself from those people once I recognize who those people are. They might not say to me, but I may hear something. I heard this person say that somebody got this job opportunity because they were a Black woman.”*

In such instances, the coping is playing out in the form of retreating from situations, people, or contexts, where a racial-, gender-, or religion-based microaggression is perceived. Sometimes the dissociation is not so much in terms of association with people but is expressed as subtle forms of retreating, reframing, or changing the subject in the case of particular conversations that are uncomfortable, as the following excerpt illustrates:

*“A boss at work once—I was single and we were out at lunch. We had a pretty good relationship, but it was a work relationship, and she knew that I was single and she even said something about a guy in a restaurant, like, oh, he looks like your type, just assuming that I would want to date a male. Like why would that even come up? Why is that assumption we make that the majority of the population would be in that way? It would be nice if we would just stop making these assumptions because it felt uncomfortable. I didn’t say anything at that point in time. I just kind of changed the subject because that’s just really uncomfortable. But then it keeps me in this weird tense state. It’s like I never really feel like I can be myself. And I feel like an imposter because no one even knows. No one even knows, and that’s uncomfortable.”*

Another form of retreating or disassociation was in terms of relegating a particular identity to the background in the face of a perceived microaggression to that identity. For a woman candidate approaching an interview and hoping to avoid a gendered microaggression that might question her professional commitment, owing to her nonwork roles, a way of coping or forestalling the occurrence of a microaggression emerged in the form of dissociating, albeit temporarily, from a particular identity.

*“I’ve even thought of maybe I should take off my wedding ring when I’m being interviewed. Maybe they’re going to be wondering if I have children or what my home life is. How many years do I want to work?”*

With regard to gender-based microaggressions, one participant noted their inability to come back at a gendered microaggression, other than framing a response in her own mind.

*“There was this one instance where we were at a robotics meet and like some guy came up to me and like, he asked me like some really weird sexual question and I was like excuse me. And then he like changed it. And I was like, ask me something relevant to robotics. It made me really uncomfortable, but again, like, I couldn’t really vocalize it because that was a male-dominated space. And like, this was somebody I just met for the first time. So this is like how do you exactly respond to this? It kinda hurt because like I was captain of the team and I like worked hard to get there and like bring my team and everything. It’s just… why is that still your first reaction when you see me, rather than seeing me as a captain, you see me as a woman first!”*

Instances such as the gendered microaggression noted in the above excerpt highlight the scope for mentoring of women in spaces where they may not be adequately represented. Given the question that this participant noted about not knowing how she ought to respond to a sexually laden comment in a professional space, support groups such as women in STEM can be a source of shared learning for coping with such microaggressions.

#### 3.1.3. Coping by Rejecting or Stonewalling

Coping by rejecting or stonewalling is an extension of the earlier approach to coping by avoidance or disassociation, in which the recipient of the microaggression engages in actively stonewalling or guarding and protecting their marginalized identities from further oppression.

The following excerpt highlights an attempt to create a barrier by putting on a self-defensive front of hyper-masculinity in order to shield a racial identity from perceived slights.

*“That’s just a daily endeavor. But I’m growing up and dealing with stereotypes of dealing with a microaggression and it leaves a chip on my shoulder and then it leaves me to be guarded all the time because I feel like if I am too vulnerable amongst people, then they may see a chink in my armor and possibly feel that they can say something that’s not right or they say that it is discriminatory.* *So I’d rather… this sounds bad, but I’m, I’ve started to like put on a very stoic and slightly, slightly hyper-masculine and intimidating factor. Not necessarily I’m going out to intimidate somebody, but I, I am putting on this stoic and a hyper-masculine self in order for people to understand you can’t mess with me. You can’t talk to me a type of way. No, you can’t touch my hair.”*

One of our participants noted the environmental microaggression of having to deal with policing in grocery aisles owing to their racial identity and the associated stereotype of being perceived as a safety risk for items in grocery stores. Her way of countering such forms of microaggressions was to be on guard all the time, as she notes:

*“It’s just feeling much more on guard. I think that I’m being very deliberate. Like, okay, I’m touching this and I’m holding it here. It’s going into a basket. Like I’m not putting anything into my hoodie. Cause it’s also accessible. Just hold this. And then I check out with it, like something very clear, like, okay, this is going, you can see that it is in this space and it’s not going into anything. And so it’s just being much more, yeah, just much more deliberate in my actions and what I’m doing to try and create some realm of safe space for everyone.”*

This notion of defending and guarding against a further threat by way of yet another microaggression was summed up by the following quote:

*“I’m super sensitive to this conversation, therefore I’m rubbed the wrong way. I automatically do. I know like coming in, I have a wall up as far as who I can be, or how open I can be.”*

Such mechanisms of stonewalling or shielding oppressed identities can be further augmented through mentoring for anticipating common microaggressions and ways to counter them. Potential ways of deflecting or diffusing situations where a microaggression is known to operate can also be modeled by mentors to aid in coping. Even at the level of awareness building, to be sensitized to potential microaggressions, mentors can play a role in helping minority groups be better prepared for receiving and shielding against known microaggressions.

#### 3.1.4. Coping by Restraining and Internalizing

This form of coping refers to holding it in and bottling up the resentment or reactions that may otherwise be expressed by responding in some form. It is a mechanism of either ignoring, letting pass, or reining in any negative emotions or reactions that emerge due to the microaggression. Individuals engaging in this form of coping are internalizing the impact and attempting to deescalate the situation where a microaggression plays out.

This approach of seething within but playing it calm or seemingly ignoring the microaggression is characterized by restraint, following an appraisal of the microaggression and potential harm that may be wrought by responding in any other way. We have heard of such approaches in the context of racial microaggressions, manifesting in Black people being subject to additional checks, such as being pulled aside by cops in traffic or additional screenings at airports or even policing in grocery aisles. This is how the coping was articulated:

*“Yeah, I mean if you react then like you get in trouble, right? I know better than to just completely react, because it will only end negatively for me. Oh, especially outside of… at least in school its sort of an isolated environment, but in the real world… yeah right! You can’t be Black and acting crazy. You’d be killed! Are you serious?”*

*“Cause usually there’s nothing you can do. Because the reality is if you react equally, it’s always going to be, Oh, this angry Black woman reacted! Or, or, you know, in these days where every other month there’s some police officer killing someone, it’s like, I don’t want to die just because this person is an idiot. So you just kind of let it go. But otherwise, what are you going to do? Is it worth me potentially facing greater consequences to correct this one person than to just let them say their foolishness or whatever and keep it moving.”*

*“I generally try to ignore it. I don’t want to escalate a situation because of how quickly things can escalate. I’m like, I don’t want to make this potentially worse when it doesn’t need to do so. So I’d rather just ignore it, do my thing, and walk out.”*

On the face of it, while responding to a microaggression in this way appears to leave no impact on the recipient because there is a seeming nonchalance in the response, in actuality, all respondents spoke about the simmering resentment and hurt they carried in the face of microaggressions, where rebutting or countering a microaggression appeared to be impossible or detrimental to their physical and mental health. The only coping in this instance happens by bottling up the feelings and internalizing the impact of the microaggression. Because such a mechanism of coping leaves little room for venting or airing the grievances emerging from the exclusion entailed in the microaggression, if left to simmer, the resentment, helplessness, and negative effect of the microaggression can be damaging to the psyche of the individuals experiencing such a microaggression. It is here that mentoring can be particularly useful to help process the negative effects and facilitate a means of returning some form of agency to the individual experiencing powerlessness in the course of a microaggression.

#### 3.1.5. Coping by Seeking Support and Reconnecting (with Safe Spaces)

Such a coping mechanism entails an active attempt on the part of the receiver of the microaggression to seek and receive support from allies such as friends, peers, mentors, or support groups or even finding safe spaces by engaging in actions like journaling to cope with a microaggression experience. Unlike the earlier approach of bottling it in, this form of dealing with a microaggression involves an unburdening by talking about the experience with others who can serve as sounding boards or offer support to cope or even chronicling the experience in journals to help process the feelings. The turn to such safe spaces for coping is evidenced in some of the following excerpts:

*“Where there are conversations that need to be had, that aren’t being had… usually you have people that come in and… they call them safe spaces… where people come in like all Black people, all women or all LGBTQ—they come in and talk about their plights, they come in and talk about their problems and get everything off their chest.”*

Without calling them safe spaces, some spoke about seeking out other individuals who shared similar experiences to talk through their own experiences:

*“We talk about the struggles of being Black, the invisible weight of being Black, of walking around in Black skin is automatically a weight that’s put on you, because you know you have to act different, in this day and age, to stay alive.”*

Reflecting on hearing a disparaging comment in relation to their race, one participant noted:

*“It was like it sucks; it was insulting. It’s like I worked my way here, right? I mean I talked about it with a couple of friends that were there at the house. I’m like, well this just happened.”*

*“At that point I talked to my friends here. I asked them like, okay, is this normal?”*

While most of the support seeking happened through finding kinship with similar affinity groups, for some, the turn to seek safe spaces was by either journaling or posting on social media to share their experiences.

*“Any traumatizing experience for me, I write a personal journal, I like. I write down how I am feeling, why it may happen, what I wanna do about it, how am I feeling right now and how should I feel. Is what I’m feeling irrational, is what I’m feeling not fair. So I usually find my journal to get my thoughts down.”*

*“This time I posted on social media. Like I posted, ‘Wow, I can’t believe that I was called the N word today!’ I put that on Facebook.”*

Another form of seeking support was articulated by turning to spirituality or religion as an anchor to help cope with the aftermath of a microaggression.

*“Sometimes I feel angry, but in Buddhism, I believe in changing my mind, internally, to make myself not be angry, just let it go.”*

For microaggressions that are religion-based and involve a perceived attack on one’s faith, another mechanism was to turn to the religion itself and seek out support from within the faith to help work through the microaggression.

*“And like first not knowing like how to respond, but then like doing research and like learning and speaking to priests. I went and I talked to a couple of the priests. And I talked to them like, you know, what could I do?”*

When individuals recognize they need to receive support through talking with friends, posting on social media, or finding other safe spaces of affinity groups, such an approach to coping is actively seeking out mentors. Very often, it is peer mentoring that can help affirm the experience of marginalization and help with coping. The need for other mentoring models, such as faith leaders to turn to or mirrored minority representation in leadership positions, can help mitigate the effect of microaggressions, as also highlighted in this comment: *“It makes me feel good to know that I had minority faculty and staff that are here as well, as another support system.”*

#### 3.1.6. Coping by Redoubling (Effort)

Another way in which some individuals address a microaggression is by redoubling their efforts in their area of work so that their work and competence speak for themselves rather than their marginalized identity that is being targeted by a microaggression. Thus, the mechanism of coping is in the form of ensuing behavior that focuses on proving the assumptions driving the microaggression wrong or overcompensating via their work. Here are some quotes that describe this approach to coping:

*“But I do feel pressured to perform due to my ethnicity and the stereotypes that may be tied to my ethnicity. And then, I also feel like sometimes, I have to ask for more responsibility or take on more of leadership positions.”*

*“When it happens (gendered racial microaggression), it makes me work twice as hard because I wanna be the person that my boss thinks of as having worked harder to get to where I am. It just makes me more determined to work harder and prove myself.”*

With microaggressions that disparaged a women’s ability to perform on the job or even in the area of sports, where there is an implicit assumption of lesser capability in relation to men, the microaggression experience has prompted some women to be more motivated to succeed and prove their competence.

*“I’m always trying to prove a point, that girls can do anything that boys can do, maybe even better”*

*“I’ll go to open hockey with some friends and I’ll be like the only girl. And it’s just like the way the guys react to me is very different. Most of the time, they wouldn’t tackle me…they’ll just ignore me…they won’t really try to stop me or actually play with me as a normal player… I feel like I’m not good enough for them to even try and compete against… it feels like a dismissal… no matter how many good moves I have. And I feel like I have more to prove, like I have to work even harder, and even if I’m better than like half the guys out there, which most of the time I am better than a whole lot of them, I have to fight a lot. It usually just makes me work harder. I just try and show them that I can keep up with them and I can play.”*

Attempting to compensate for the dismissal that a microaggression relays can, thus, be one way of seeking to neutralize the aggression. It is essentially attempting to turn around the dismissal or rebuke packaged in the microaggression and invalidate it on the grounds of merit and performance. While such an approach might come easily to some individuals or be more amenable in certain situations, active mentoring can help neutralize microaggressions by channeling the hurt in such ways as to invalidate the microaggression itself, which can be a potential area for coaching. Mentoring can, thus, help in finding opportunities for similar applications of converting a negative effect into a positive outcome by way of alternate behavior.

## 4. Enabling Coping: Emergent Mentoring Linkages

In considering the different coping mechanisms discussed above, we found possible linkages to mentoring for all the coping strategies identified in our study, as discussed in the earlier section. The emergent pathways to mentoring appear as extensions of the coping strategies, which is in line with the idea of mentoring as enactment [41]. In this section, we present a framework for integrating mentoring with coping for managing the microaggression experience.

In reviewing the research and theory of mentoring, Bozeman and Feeney [42] explored the multiple definitions offered for the concept of mentoring, depending on the formality or informality of the relationship and the number and types of parties involved. Most definitions, however, reflect Kram’s [43] focus on more senior individual(s) who use their influence and experience to help advance others. Kram’s seminal work [43,44] also describes two core functions of mentoring that have informed much of the research in the area to date: career support and psychosocial support. Career support aspects are instrumental in career development and include providing mentees with exposure, visibility, protection and access, sponsorship, and coaching. Psychosocial elements have more emotional foci and include role modeling, acceptance and confirmation, counseling, and friendship.

Research suggests that effective engagement in these activities offers benefits to those who have access to mentor relationships, leading to higher reported salaries, increased promotion rates, greater career satisfaction, higher organizational commitment, less intention to leave an organization, and lower levels of turnover [45,46,47,48,49,50,51,52,53] on the career-support side and positive behavioral, attitudinal, motivational, and relational outcomes [54] on the psychosocial-support end. Thus mentoring seems a likely tool to employ in efforts to address the marginalizing effects of microaggressions. Table 1 offers linkages between coping mechanisms to microaggressions employed by sample participants and the various functions and domains of mentoring. Gandhi and Johnson [55] offer quantitative and qualitative evidence that attention to best practices, tools, and techniques in such mentorship training for HIV researchers leads to enhanced competencies in six mentorship domains: communication, expectation alignment, assessment of understanding, fostering independence, addressing diversity and promoting development. These domains were examined in the context of mentorship functions suggested by study participant responses.

As this study took place at a major mid-Atlantic research university, undergraduate and graduate respondents are mentored formally and informally by peers, advisors, faculty members, corporate supporters, and others in both career development and psychosocial domains. Respondents actively resisting microaggressions through their voices may benefit from mentor efforts that enhance communication skills and patterns, targeted to making others aware of the bias being demonstrated by microaggressions. Mentor suggestions on how best to “read” an audience when delivering information that could potentially evoke a conflict between parties would assist the mentee in educating others on the microaggression. For example, the race-based microaggression presented in the table was noted by one participant as offering the potential for an escalation of exchanges between parties without proper thought given to the initial response.

Those who tend to cope with microaggressions through withdrawal may begin to address their feelings of alienation and isolation through techniques that aid the mentee in a deeper understanding of the nature of and response to the microaggression felt. In these situations, mentors may want to assist their mentees in using communication techniques, such as effectively conveying to others the deep-seated commitment felt towards the basic tenets of a religion, regardless of whether others share the same loyalty.

When mentees “put up walls” to protect themselves from being “rubbed the wrong way” and feel belittled, frustrated, invisible, or powerless, mentors can focus their efforts on assisting mentees in understanding the impetus for the microaggression as a foundation for communicating the nature of the microaggression experienced to the other party, as well as to begin work on an acceptance of diverse views and positions.

Approaching microaggressions through internalizing the anger, anxiety, rage, or fear felt in the situation can certainly result in both mental and physical stress and overall negative consequences over time. Mentor support tools that offer ways to promote mentee development of alternative responses and foster the value of holding views that are independent of others are key.

Perhaps the most frequently recognized role of mentors is that of offering support. Those who cope with microaggressions by finding “safe spaces” are well served by mentors who serve as “sounding boards” and share ideas in groups that foster support for those who identify with marginalized groups.

Those who cope with microaggressions through a redoubling of effort, as evidenced by the participant who felt pressure to perform for their ethnicity, face the potential of physical or mental burnout. Accordingly, mentors may work with the mentees to develop an understanding that the mentee is not responsible for their entire affinity group and develop techniques like goal-setting, which better aligns the expectations of all involved parties.

Table 2 offers a bridge between research and practice within the specific context of a diversity and inclusion program being developed in response to a diversity and inclusion training initiative for faculty, staff, and students in a college of a major mid-Atlantic research university. Specific programming to be covered in that training, informed by the coping mechanisms used by those who are marginalized by microaggressions, is offered in order to begin building an inclusive environment for all.

Specifically, those individuals who actively resist microaggressions can best be supported by mentors who recognize that using their voice can lead to escalation and disputes between the involved parties. Accordingly, dispute resolution processes and procedures can be covered during mentor training, as well as focusing on audience analysis in the communication area, how to hold difficult conversations, and how to give and get feedback in an escalating verbal situation.

Those who cope by withdrawing need mentors who are trained in helping mentees to develop enhanced self-awareness skills, including a focus on self-efficacy. Ways for mentees to begin communication can be facilitated by those who can help them identify viable communication styles, given knowledge of their relevant audiences. Mentors themselves must recognize their obligation as role models and must work to help the mentee identify support groups consistent with the marginalized identity.

Active rejection of the microaggression as a coping mechanism, through stonewalling, offers the mentor an opportunity to counsel the mentee on implicit bias and emotional intelligence [57], which are two core training topics that underlie most of the mechanisms. Both of these topics should be featured in any mentor training program.

Internalization as a regular coping mechanism can produce both mental and physical issues. Mentor training should focus on communication techniques that allow self-awareness items, such as self-esteem, to be drawn out of the mentee. Training can also focus on managing and regulating negative emotions, a component of emotional intelligence [58]. These techniques are further expanded for those who cope with microaggressions through support or safe spaces. Training in communication techniques, such as journaling, is recommended. From the mentor perspective, honing listening techniques to serve more effectively as a sounding board is key, as is understanding the nature of support groups to aid the marginalized individual.

Finally, those who cope with microaggressions by redoubling their efforts face potential burnout [58] from both a mental and physical standpoint. Accordingly, training mentors in organization skills, such as goal setting tactics that can be shared with mentees, is important, as well as developing communication skills that help the mentee identify and align their expectations.

**Figure 1:**
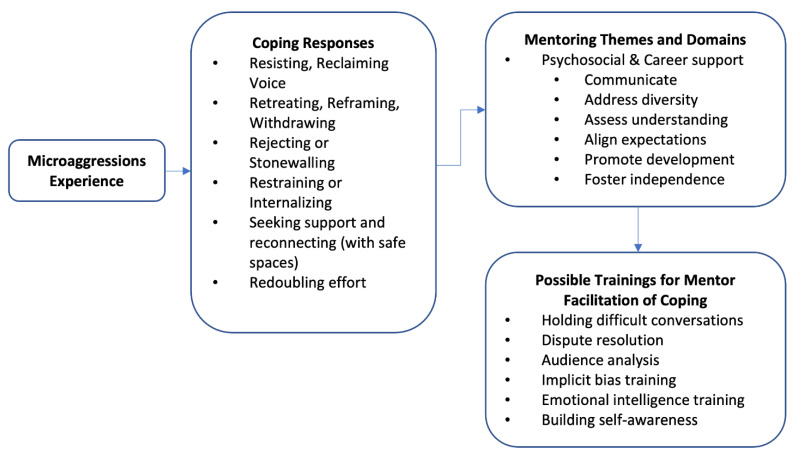
Coping mechanisms and forwarded mentoring linkages.

Thus, as depicted in Figure 1, it is emergent that the coping strategies identified in our study can be linked to mentoring functions and domains, with suggested training to facilitate the processes of coping in the aftermath of a microaggression experience.

## 5. Discussion

When facing microaggressions, there can be a variety of ways in which people respond and, hence, cope with both the microaggression itself and their own marginalized identities. Our analysis of microaggression experiences of different marginalized identities and the coping mechanisms employed by recipients of the microaggression has revealed some underlying patterns. We classify the different coping mechanisms as coping by (a) resisting or reclaiming voice, (b) retreating, reframing, or withdrawing, (c) rejecting or stonewalling, (d) restraining and internalizing, (e) seeking support and reconnecting (with safe spaces), and (f) redoubling (effort). Some of the coping mechanisms offer a form of pushback, whether in terms of actively resisting the microaggression by calling out the exclusionary and discriminatory behavior entailed in the microaggression or by seeking to invalidate the microaggression by letting one’s actions and behavior speak, in terms of redoubling effort, to counter the very premise of the microaggression. Other forms involve some form of a self-defense mechanism to cope, including stonewalling, retreating, or dissociating from the source of the microaggression or one’s targeted identity or restraining oneself from responding to the microaggression in the interest of self-preservation. In such instances though, residues of the experience remain, and there is a need to process the negative impact of the microaggression, either cognitively or emotively. It is here that mentoring can play a particularly important role. Even in coping mechanisms characterized by actively seeking support and safe spaces, mentoring offers a natural avenue by offering a path to safe spaces. For each of the different coping strategies, we offer ways that mentoring can facilitate the coping process.

Mentoring is an approach that can be employed in dealing with the different microaggression coping mechanisms. Further, formal mentor training can better support mentees in giving a voice to the microaggressions exercised against them by making organizations members, as a whole, and mentors specifically focus on understanding the nature and incidence of implicit bias. Workshops defining the concept and offering facilitated discussions of how to recognize and address such biases can raise competency skill scores in the area [59].

Advocating mentoring programs as a means to address diversity and inclusion efforts, such as recognizing microaggressions, is incomplete without attention to the nature and structure of implemented actions that comprise an organization’s formal mentoring program. Relying on the seminal work of Levinson, Darrow, Klein, Levinson, and McKee [60] and Kram [44,61], the current view of mentoring offers a network focus, with developmental input from multiple sources. Such variability introduces an opportunity for a spectrum of mentorship techniques and outcomes to arise [62]. Accordingly, formal mentorship training should be offered by those organizations with such programming in place, especially in mentoring situations where microaggressions may be the focus. Pfund and colleagues [59] have found that trained mentors are more likely than untrained mentors to consider issues of diversity and work more effectively with mentees. Gandhi and Johnson [55] offered quantitative and qualitative evidence that attention to best practices, tools, and techniques in such mentorship training for HIV researchers leads to enhanced competencies in communication, expectation alignment, assessment of understanding, fostering independence, addressing diversity, and promoting development. Thus, mentoring to facilitate coping in the light of microaggressions can benefit from mentorship training, as suggested earlier.

In coping with microaggressions, we have forwarded some linkages to mentoring that are in line with the view of mentoring as “enactments” [41], with mentor behaviors such as providing reassurance, asking questions, and giving feedback linked to psychological safety. Such enactments provided by mentors can be particularly useful to help facilitate sense-making and cope with microaggressions as well. For example, in developing a mentorship program addressing microaggressions, where coping is through retreating or withdrawal, special attention must be paid to mentor access. Those that withdraw may lack self-awareness, efficacy, and esteem. Accordingly, mentors who model such roles and the accompanying skills and competencies will assist in developing such traits. However, Ragins and Kram [62] and Blake-Beard, Murrell, and Thomas [45] noted the challenges of accessing mentors for women and individuals of color as limited numbers of role models often exist in organizations because of “glass ceilings” or “concrete ceilings”. Role modeling is a key element in mentoring, providing a means by which the mentee can identify with the valued organizational norms and attributes. In the absence of such exemplars, the skills and competencies needed for success in the organization may be limited in their development. For each of the coping mechanisms emergent in our study, we have highlighted possible mentoring domains and mentoring enactments for psychosocial and career support, whether it be dispute resolution and holding difficult conversations to fuel processes of reclaiming one’s voice and resisting microaggressions or aligning expectations and modeling goal-oriented behaviors for redoubling efforts in the face of a microaggression.

## 6. Conclusions

The experience of exclusion and slights communicated by a microaggression experience can leave recipients of the microaggression with a plethora of negative emotions. Responses to the microaggression also vary. Our research on the microaggression experiences of different marginalized identities has revealed different coping mechanisms or strategies employed in the face of a microaggression. We indicate ways in which mentoring can help with either ameliorating the impact, processing the experience, or even countering and responding to the microaggression in appropriate ways. Thus, in the paper, we highlight specific coping mechanisms for dealing with microaggressions and offer ways to facilitate the process through mentoring.

## Figures and Tables

**Table 1:** Linkages between microaggression responses, coping mechanisms, and mentoring functions and domains.

Microaggression Responses ^i^	Coping Mechanisms	Mentoring Function and Some Related Excerpts	Mentoring Domains ^ii^
Self-doubt, anger, guilt, belittlement, alienation, isolation, depression, anxiety, frustration, powerlessness, invisibility, loss of integrity, rage, fear	Resisting/Reclaiming Voice	**Career Support** *“* *I think I can remember a situation at my last job where somebody assumed a stereotype, I forgot what the context was, but was assuming a stereotype about Black people and I chose to tell them like, listen, that’s not the case. Like you need to widen your understanding of that.* **Psychosocial** *“If a person doesn’t have any verbal restraint when they were talking to me, then I don’t have to hold my punch. So somebody says, oh, you like watermelon? I could say no I don’t! I really don’t, but it’s just like I’m direct with it. Using a very quick, succinct, NO, I don’t like that or, no, I don’t really subscribe to that.”*	Address diversity Communicate
	Retreating, Reframing, Withdrawing	**Career Support** *“I’ve even thought of maybe I should take off my wedding ring when I’m being interviewed. Maybe they’re going to be wondering if I have children or what my home life is. How many years do I want to work?”* **Psychosocial** *“Like when they’re talking about religion, I never participate in the conversation.”*	Assess understanding Align expectations Address diversity
	Rejecting or Stonewalling	**Psychosocial** *“I’m super sensitive to this conversation, therefore, I’m rubbed the wrong way. I automatically do. I know like coming in, I have a wall up as far as who I can be, or how open I can be.”*	Assess understanding Communication
	Restraining and Internalizing	**Psychosocial** *“So you just kind of let it go. But otherwise, what are you going to do? Is it worth me potentially facing greater consequences to correct this one person than to just let them say their foolishness or whatever and keep it moving.”*	Promote development Foster independence
	Seeking Support and Reconnecting (with safe spaces)	**Psychosocial** *“* *Where there are conversations that need to be had, that aren’t being had… usually you have people that come in and… they call them safe spaces… where people come in like all Black people, all women or all LGBTQ—they come in and talk about their plights, they come in and talk about their problems and get everything off their chest.”*	Communicate Foster independence
	Redoubling (efforts)	**Career Support** *“But I do feel pressured to perform, uh, due to my ethnicity and the stereotypes that may be tied to my ethnicity. And then I also feel like sometimes I have to ask for more responsibility or take on more of leadership positions” “When it happens (gendered racial microaggression), it makes me work twice as hard because I wanna be the person that my boss thinks of as having worked harder to get to where I am. It just makes me more determined to work harder and prove myself.”*	Address diversity Align expectations

^i^ [56]; ^ii^ [55].

**Table 2:** Mentor training session topics to support microaggression coping mechanisms.

Coping Mechanisms	Mentoring Domains ^ii^	Training Topic
Resisting/Reclaiming Voice	Address diversity Communicate	Dispute resolution techniques
		Communication skills: Audience analysis
		Holding difficult conversations
		Giving/getting feedback
Retreating, Reframing, Withdrawing	Assess understanding Align expectations Address diversity	Self-awareness tools: Self-efficacy
		Communication skills: Audience analysis
		Communication style identification
		Mentor: Role model
		Support group identification
Rejecting or Stonewalling	Assess understanding Communication	Implicit bias training
		Emotional intelligence training
Restraining and Internalizing	Promote development Foster independence	Communication skills: Self-awareness: self-esteem
		Emotional intelligence training
Seeking Support and Reconnecting (with safe spaces)	Communicate Foster independence	Communication skills: Active listening
		Communication tools: Journaling
Redoubling (efforts)	Address diversity Align expectations	Organization skills: Goal setting
		Communication skills: Aligning expectation

^ii^ [55].

## Data Availability

The data presented in this study are available on request from the corresponding author. The data are not publicly available due to privacy and confidentiality restrictions.
